# In Situ Modification of Reverse Osmosis Membrane Elements for Enhanced Removal of Multiple Micropollutants

**DOI:** 10.3390/membranes9020028

**Published:** 2019-02-13

**Authors:** Katie Baransi-Karkaby, Maria Bass, Viatcheslav Freger

**Affiliations:** 1 Technion—Israel Institute of Technology, Wolfson Department of Chemical Engineering, Technion City, Haifa 32000, Israel; bass@technion.ac.il; 2 The Galilee Society, Institute of Applied Research, P.O. Box 437, Shefa-amr 20200, Israel

**Keywords:** in situ membrane modification, micropollutant removal, boron removal, spiral wound elements, reverse osmosis

## Abstract

Reverse osmosis (RO) membranes are widely used for desalination and water treatment. However, they insufficiently reject some small uncharged micropollutants, such as certain endocrine-disrupting, pharmaceutically active compounds and boric acid, increasingly present in water sources and wastewater. This study examines the feasibility of improving rejection of multiple micropollutants in commercial low-pressure RO membrane elements using concentration polarization- and surfactant-enhanced surface polymerization. Low-pressure membrane elements modified by grafting poly(glycidyl methacrylate) showed enhanced rejection of all tested solutes (model organic micropollutants, boric acid, and NaCl), with permeability somewhat reduced, but comparable with commercial brackish water RO membranes. The study demonstrates the potential and up-scalability of grafting as an in situ method for improving removal of various classes of organic and inorganic micropollutants and tuning performance in RO and other dense composite membranes for water purification.

## 1. Introduction

The presence of organic micropollutants, in particular, endocrine-disrupting compounds (EDCs) and pharmaceutically active compounds (PhACs), in sewage treatment plant effluents emerged as an issue of growing concern over recent years [1,2,3,4,5,6]. This is an extremely diverse group of substances that include industrial chemicals, agrochemicals, pharmaceuticals, hormones, etc. that can have adverse effects on ecological systems and human health [7,8]. Many micropollutants tend to persist or only partially degrade in conventional wastewater treatment, thereby tending to accumulate in recreational and drinking water sources [1,9,10,11]. Up to date, there are no regulatory requirements for monitoring EDCs and PhACs in drinking water [1], yet there are attempts to limit their entrance.

Nanofiltration (NF) and reverse osmosis (RO) membrane processes offer an attractive solution for improving water quality by removing organic contaminants to a greater extent than conventional water treatment plants [11] or concentrating them in smaller reject volumes that may be easier to treat and safely dispose of. However, the rejection of small organic compounds by membrane filtration is strongly influenced by physicochemical properties of the compounds (e.g., molecular size, solubility, hydrophobicity, charge); therefore, the removal of some organics by membrane processes can be low. Notably, studies of organic removal by commercial RO and NF membranes in pilot and full-scale installations showed contradictory results. Many show a high rejection of micropollutants by membranes [1,11,12,13,14]; however, others report incomplete removal by RO and NF, which may also greatly vary between different membranes and specific pollutants [15,16,17,18]. Moreover, sorption of organics within membranes and filtration systems is a phenomenon that may overestimate the true rejection of strongly adsorbed solutes, if the steady state is not reached [5,19,20,21].

Sorption of organics within the membrane itself and, especially, the active layer, as result of affinity toward the polyamide, also enhances permeation and lowers rejection of small uncharged organics in RO and NF [2,6,22,23,24,25]. The undesired sorption and permeation of uncharged organics is exacerbated by the fact that they are unaffected by electrostatic exclusion mechanisms (Donnan and dielectric) that are responsible for the high salt rejection in RO and NF [26,27,28,29,30]. The rejection of uncharged micropollutants is then controlled mostly by the affinity, size exclusion, and molecular friction [6,22,23]. Similar factors are responsible for the low rejection of boric acid, an important inorganic micropollutant in seawater desalination. Boric acid is naturally present in sea water and aquifers (>4 mg/L) and can also be introduced in waste water from anthropogenic sources [31,32,33]. High concentration of boric acid in irrigation water accelerates plant decay and expiration, yet it is insufficiently removed by RO membranes at pH below its pKa ~8.6 (in artificial seawater), when boric acid is uncharged, which incurs additional costs in sea and brackish water desalination [31,34].

Surface modification of NF/RO membranes by graft polymerization was shown to be an appealing way to improve micropollutant removal [33,35,36,37,38]. The concentration polarization (CP)-enhanced procedure, whereby a mixture of suitable monomers and initiators is simply filtered through the membrane, resulting in enhanced polymerization at its surface, offers a particularly facile way to form a modifying coating layer on top of the membrane and minimize monomer consumption [35,38]. Moreover, our group demonstrated recently that the monomer consumption may be further reduced and coating uniformity can be improved by adding a non-ionic surfactant such as Triton X100 to the modifying solution, which solubilizes the monomer within surfactant micelles. Since micelles concentrate the hydrophobic monomer and undergo a stronger CP, this helps bringing the monomer to the surface and facilitates surface grafting [39]. 

Unfortunately, most previous studies were performed at the laboratory scale, with very few reports involving feasibility tests up-scaled to commercial elements [38]. The potential of up-scaled in situ modification for improving micropollutant removal in genuine commercial elements remains largely unexplored. Therefore, in this study, we examine the feasibility of in situ surfactant-enhanced surface modification up-scaled to commercial membrane elements for improved micropollutant removal. Since, from a practical standpoint, tuning the modification recipe to each group of pollutants would not be attractive, another point examined here is whether a single optimal procedure can improve rejection of a range of micropollutants that belong to different classes and differ in their physicochemical characteristics. The focus is on low-pressure reverse osmosis (LPRO), which is particularly attractive for water treatment due to high permeability, relatively high selectivity, and low energy consumption.

## 2. Materials and Methods

### 2.1. Materials

All chemicals used in the study were purchased from Sigma-Aldrich (Rehovot, Israel), and used without purification. The fully aromatic polyamide LPRO elements (ESPA1-2521, membrane active area 9200 cm^2^ [40]) were purchased from Hydranautics (Oceanside, CA). Deionized water (DW) was used for preparing solutions in all experiments.

Bisphenol-A (BPA), classified as an EDC, and carbamazepine (CBZ) and acetaminophen (ACN), classified as PhACs, were used as model micropollutants for membrane performance tests, along with boric acid, an inorganic micropollutant. These contaminants were selected based on their occurrence in water sources, and relatively poor rejection by RO membranes. Table 1 summarizes their structure and physicochemical characteristics. The pK_a_ values, as well as that of boric acid (determined to be pK_a_ ~9.2 in fresh water [31]), indicate that all tested micropollutants are uncharged at neutral pH.

### 2.2. Membrane Modification

**Figure 1:**
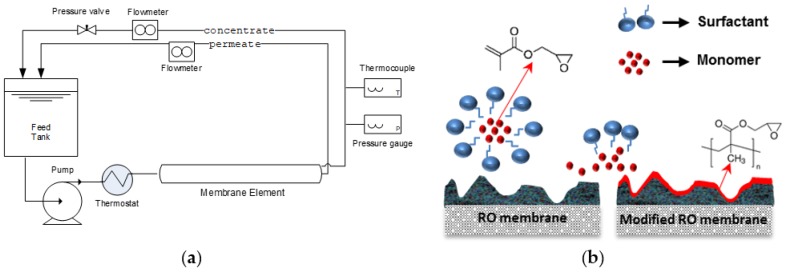
(**a**) Schematic representation of the set-ups for modification and the testing element. (**b**) Schematic illustration of formation mechanism of the grafted layer.

Modification of ESPA1-2521 elements using a CP-enhanced grafting procedure with added surfactant [39] was performed in a cross-flow filtration mode. The set-up included an 8-L feed tank, the membrane module, a high-pressure pump with a pulsation damper for minimizing pressure fluctuations in the system, a heat exchanger, a valve for pressure regulation, and two flow meters installed in the concentrate and permeate lines that were used for monitoring the cross-flow rate and adjusting recovery. The system was operated in a close loop with permeate and concentrate streams recirculated back to the feed tank (see Figure 1a).

The modifying solution initially contained 2 mM glycidyl methacrylate (GMA) monomer, and 0.045 mM surfactant Triton X-100. The solution was first filtered through the membrane for 5 min at the feed pressure of 20 bar in order to adjust recovery. Thereafter, the cross-linker (*N*,*N*′-methylene-bisacrylamide, MBAA) and initiators potassium persulfate and potassium metabisulfite, separately dissolved in DW, were added to the modifying solution; their concentrations in the modification solution were set to 0.003 mM, 4 mM, and 2 mM, respectively. During modification, the tangential velocity of the solution was kept small (~1.8 cm/s) in order to enhance concentration polarization of the reactants and promote the surface grafting reaction. Recovery rate was 35–45%, i.e., retentate flow was 55–65% of the feed, which allowed pushing a substantial fraction of the feed across the membrane and yet ensuring a substantial recirculation rate to avoid a strong variation of monomer concentration along the module and obtain a uniform coating. The modification was carried out for 30 min at a temperature of 24 °C. Afterward, the solution was discarded and the module was thoroughly washed with DW. Fresh DW was recirculated through the modified modules for 24 hours before the performance tests. A schematic illustration of the grafting chemistry and mechanism is shown in Figure 1b.

### 2.3. Membrane Characterization

**Figure 2:**
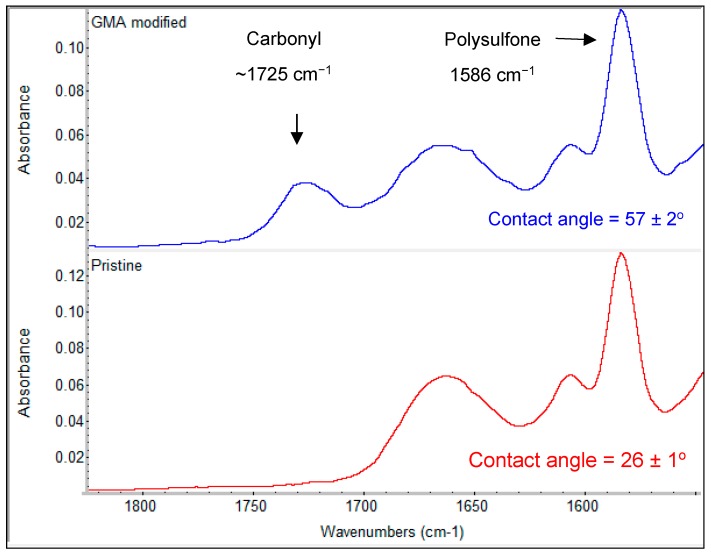
Typical spectra of a pristine ESPA1 membrane (**bottom**) and a membrane modified by grafting a poly(glycidyl methacrylate) (GMA) coating layer (**top**).

The pristine and modified ESPA1 elements were autopsied to examine changes in membrane surface characteristics. Scanning electron microscopy (SEM, Ultra-Plus FEG, Zeiss, Oberkochen, Germany) was used to image the membrane surface morphology before and after modification. Attenuated total reflection Fourier-transform infrared (ATR-FTIR) spectra were recorded as the average of 64 scans at 4-cm^−1^ resolution using a Nicolet 8700 FTIR spectrometer (Thermo-Scientific, Waltham, MA, USA) equipped with a Smart MIRacle ATR accessory with a diamond element (Pike, Madison, WI, USA). The spectra of modified membrane were recorded at different locations along and across the element in order to evaluate the uniformity of the coating. Each relative location along or across the element included 10–12 different spots. The degree of grafting (DG) was defined as

(1) $$\mathrm{DG}=\frac{A_{g}}{A_{m}},$$

where *A_g_* and *A_m_* are the areas of the IR bands characteristic to the grafted polyGMA (a carbonyl band at ~1725 cm^−1^) and the membrane (1586 cm^−1^ band characteristic of the supporting porous polysulfone layer of the original membrane), respectively, as shown in Figure 2.

Contact angle of membranes after autopsy was measured using a sessile drop of water, using a DSA 100 instrument (KRÜSS) equipped with a video camera, image grabber, and data analysis software. Every measurement was repeated at least four times (starting with a 0.25–0.45-μL drop) and reported results for each membrane sample are the average of at least five drops.

### 2.4. Membrane Testing

The filtration tests were performed on a larger pilot-scale set-up, analogous to the one used for modification (Figure 1a), but allowing higher feed flow rates, corresponding to standard operation conditions. The system included a 100-L feed tank, a membrane module housed in a high-pressure vessel, a high-pressure pump, and a pressure regulation valve. Flow meters installed in the concentrate and the permeate lines were used for monitoring the cross-flow rate and water flux. The system was operated in a closed loop with permeate, concentrate, and bypass streams recirculated back to the feed tank. 

Membrane permeability was tested by filtering DW at a constant concentrate flow rate that was set by adjusting the feed pressure between 10 and 20 bar. Two ranges of feed flow rates were used, 330–400 L/h and 600–680 L/h and the average permeability was taken. 

The NaCl and boric acid passages were measured by filtering 1500 mg/L NaCl solution or 5 mg/L boric acid solution of pH ≈ 7 at 10, 15, and 20 bar. Boron and salt passages were examined after 2 h of filtration. Then, samples were taken after 15 minutes of filtration for each pressure and recovery condition. The salt passage was determined by measuring the electric conductivity of the feed and permeate using a conductivity meter (WTW InoLab Cond 7110, Weilheim, Germany). The passage of boric acid was determined by measuring B concentration using an inductively coupled plasma (ICP) spectrometer (iCap 6000 SeriesICP-OES, Thermo Scientific, Waltham, MA, USA) at a wavelength 248 nm.

The removal of organic micropollutants was examined in the filtration tests carried out for four days at the recovery rate of 5–10%. The feed solution contained 10–12 mg/L for CBZ, 100–120 mg/L for BPA, and 100–120 mg/L for ACN at pH ≈ 7.3–7.4 (at this pH, the model solutes were uncharged). It was prepared by adding a stock solution of micropollutants in ethanol to the feed tank; the added stock solution volume did not exceed 1% of the feed volume. These micropollutant concentrations, high compared to those found in water sources and wastewater, had to be used due to the detection limit of HPLC used for analysis of the permeate. However, we believed this point was not critical for comparing pristine and modified membranes, which was our main purpose. Furthermore, except for BPA, all micropollutant concentrations were far below the solubility in water (see Table 1); therefore, no excessive non-linearity of sorption was expected, and the results and conclusions could be extrapolated to lower concentrations.

The solution containing the selected micropollutants was filtered at a feed pressure of 20 bar, and the recirculation rate was in the range of 350–680 L/h to maintain recovery within the 5–10% range. The resulting permeate flow rate was within the range 27–47 L/h for the unmodified module and 24–39 L/h for the modified module. 

The concentration of the organic micropollutants in the inlet was determined using a high-performance liquid chromatograph with an ultraviolet (UV) detector (HPLC, Agilent 1220 Infinity, Santa-Clara, CA, USA). HPLC was equipped with a Zorbax Eclipse XDB-C18 separation column; the mobile phase was a binary gradient of 0.1% formic acid in distilled deionized water (DDW) and acetonitrile at a flow rate of 1 mL/min. UV wavelengths employed for the analysis were 230 nm for BPA and CBZ, and 280 nm for ACN. Prior to HPLC injection, the feed samples were pre-filtered through 0.22-µm syringe filters made of polyvinylidene fluoride (BPA and CBZ) or polyethersulfone (ACN). 

For measuring much lower permeate concentrations, an LC equipped with a multiple stage/mass spectrometry detector unit (LC–MS/MS, Agilent 1200, Santa-Clara, CA, USA) coupled to an ion-spray interface and API 3200 triple quadrupole mass spectrometer was used. The separation column was a Purospher Star RP-18 (Merck, Darmstadt, Germany) that used a binary gradient of formic acid water solution (0.1% (*v*/*v*)) and methanol for CBZ and ACN and a binary gradient of ammonium hydroxide water solution (5 mM) and methanol for BPA. Electrospray ionization used positive ion mode for CBZ and ACN and negative ion mode for BPA. The detection limit of CBZ, ACN and BPA was 5 µg/L.

The passage (*P*) and rejection (*R*) of all tested permeants was calculated as

(2) $$P=1-R=\frac{C_{P}}{C_{F}},$$

where *C_P_* and *C_F_* are the measured permeant concentrations in the permeate and feed, respectively. Since the measured solute rejection could be affected by concentration polarization in the modules, its degree during membrane testing was estimated using the concentration polarization factor, defined as CPF = exp (*J_V_*/*k*), where *J_V_* is the measured volume flux and *k* is the mass transport coefficient. The mass transfer coefficient for pristine and modified ESPA1 elements was first estimated for NaCl (*k_NaCl_*) by measuring its passage and flux at different feed flow rates *Q*. Given that the module geometry and physical properties were constant, the standard relationship *Sh* = *ARe^n^Sc*^1/3^ [42,43,44,45], where *Sh*, *Re*, and *Sc* are the Sherwood, Reynolds, and Schmidt numbers, respectively, and *A* and *n* are constants, could be reduced to *k = A′Q^n^*, where *A′* is constant for a specific solute, and Q is the feed flow rate. The value for *k_NaCl_* was determined from measured dependence of passage on *Q* and *J_V_* using the method reported by Sutzkover et al. [44], with the value of *n* = 0.4 reported for spiral-wound elements [45]. Subsequently, the mass transfer coefficients for micropollutants were computed using the scaling relation of the boundary diffusion layer model *k* = *k_NaCl_* (*r_NaCl_*/*r_s_*)^2/3^, where the salt Stokes radius *r_NaCl_* is the average of the Stokes radii of Na^+^ and Cl^−^ ions and *r_s_* is the Stokes radius for organic solutes [46]. The value of *r_s_* was computed using the correlation between the molecular weight (Table 1) and *r_s_* [47].

## 3. Results and Discussion

### 3.1. Membrane Surface Characteristics, Morphology, and Uniformity

**Figure 3:**
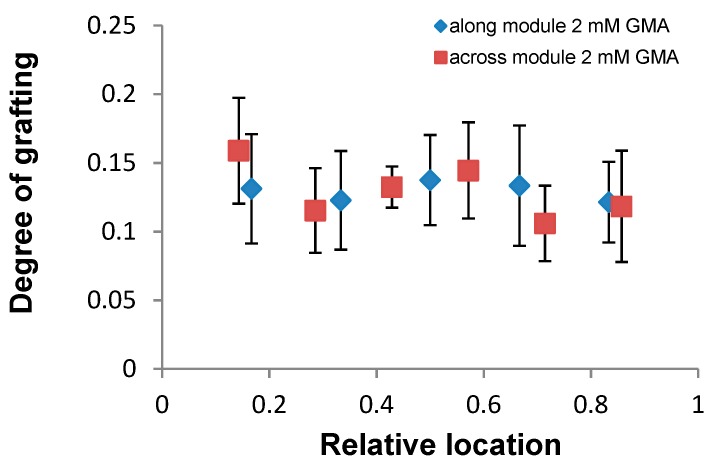
Degree of grafting (DG) of ESPA1-2521 elements modified with GMA at different locations along (entrance (0) to-exit (1)) and across (permeate edge (0) to-outer edge (1)) the element. The element was modified for 0.5 h using 2 mM GMA and 0.045 mM Triton solution. The error bars correspond to standard deviations of all DG measurements for a given location.

The grafting procedure exploited in this study was similar to the one used in a previous report [35]; however, two important modifications were made. Firstly, the surfactant (Triton X-100) was added to the modification solution, which was previously shown to facilitate grafting of GMA, reduce required monomer concentration, and improve the homogeneity of the coating [39]. Secondly, the modification was performed in cross-flow mode with 35–40% recovery, rather than dead-end mode (100% recovery) in order to overcome non-uniformity of the coating along the module [35]. The degree and uniformity of grafting was quantified after autopsy of a modified ESPA1 element and recording ATR-FTIR spectra at different locations along and across the element, relative to the entry and permeate edge (Figure 3). The results indicate that the membrane surface was successfully and uniformly modified over the entire element, while contact angle data also showed a significant increase in surface hydrophobicity. A recently developed method for the quantitative evaluation of polymer coating thickness using ATR-FTIR showed that the average DG ~0.12 corresponds to an average grafted layer thickness of about 35 nm [48].

**Figure 4:**
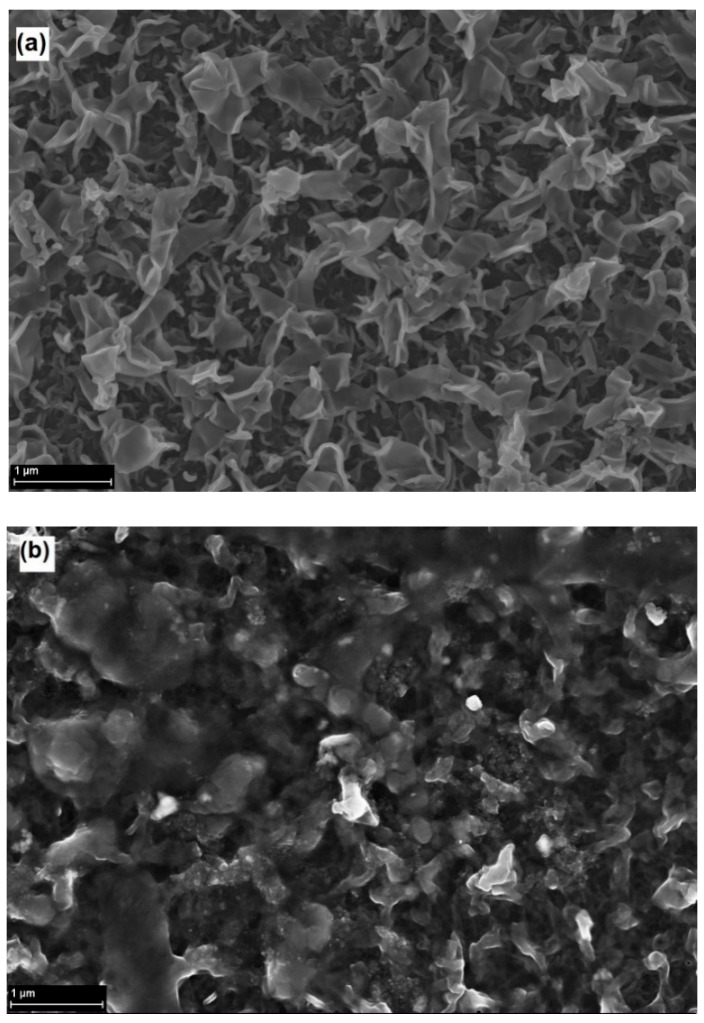
SEM images of the pristine ESPA-1 membrane (**a**), and the membrane taken from modified ESPA1-2521 module (**b**). The modification conditions were as follows: 2 mM GMA with addition of 0.045 mM Triton (0.5 h).

Figure 4 shows SEM images of the pristine and the modified ESPA1. The change in the surface morphology caused by the modification is clearly seen in Figure 4b; however, at some locations, SEM images appeared more similar to the pristine membrane, indicating that the modification was absent or too thin to change the morphology. On the other hand, each ATR-FTIR result in Figure 3 represents a much larger surface area (~3 mm^2^) compared to SEM images (~4 × 10^−5^ mm^2^). This suggests that, while the grafting is fairly uniform on the millimeter scale, the coating may vary in surface coverage and thickness on the micron scale. Nevertheless, if surface modifications mainly seal microscopic defects in the polyamide layer, as shown previously [25,33], a non-uniform coating could achieve such sealing and produce the desired improvement in performance as well. Indeed, the results presented below confirm that the obtained coating significantly improved rejection of all tested micropollutants. 

### 3.2. Membrane Performance

The selected model micropollutants significantly differed in polarity, water solubility, and molecular weight (see Table 1), especially the organic solutes compared to boric acid. Previous studies showed that many hydrophobic micropollutants tend to adsorb or dissolve within polyamide membranes and/or parts of filtration systems and produce artefacts such as superficially high non-steady-state rejection [2,5,17,19,20,21,22,36,49,50,51]. Therefore, to rule out such overestimates, it was important to verify that the examined membranes reached a steady-state performance for all examined solutes. The time required for reaching steady state was evaluated using a solution containing BPA and CBZ that was filtered through a pristine ESPA1 element for 12 days, while the pollutant concentration was monitored in the feed and permeate. During this period, the feed solution was replaced twice with a fresh one in order to speed up the equilibration. 

**Figure 5:**
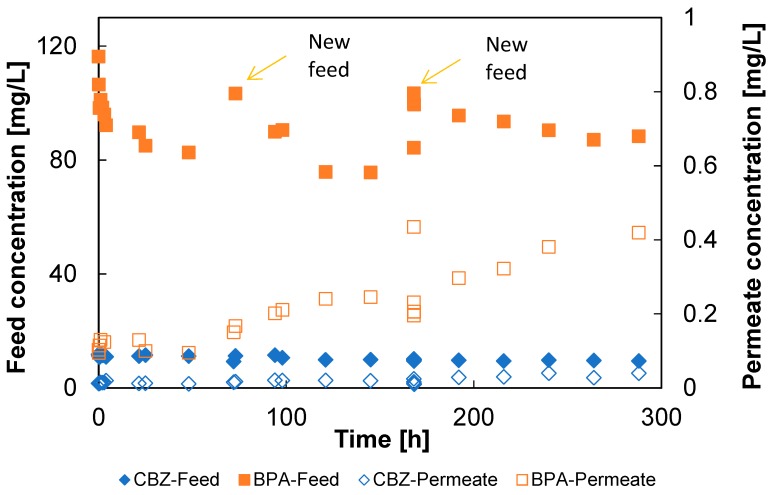
Changes in the feed and permeate concentrations of carbamazepine (CBZ) and bisphenol-A (BPA) measured with time for a pristine ESPA1-2521 element. The filled and open symbols represent the feed and permeate concentrations, respectively.

Figure 5 shows the results obtained for BPA and CBZ during the run. It is seen that the decrease in the feed concentrations of BPA and CBZ was more significant in the first 24 hours each time the feed was replaced, and then continued to change slowly up to the next replacement, while their concentrations in the permeate increased steadily, especially in the case of BPA. The results also indicate that a four-day period was about sufficient to approach the steady state; therefore, the subsequent tests were performed for this period of time.

**Figure 6:**
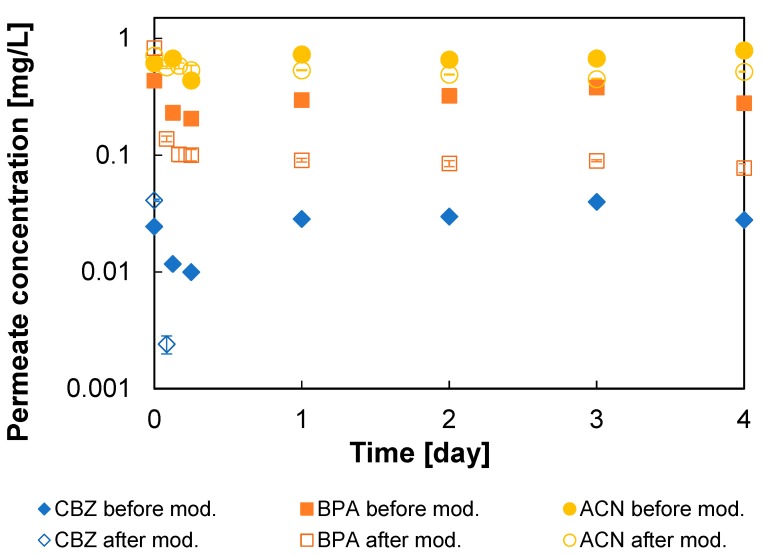
Permeate concentrations of carbamazepine (CBZ), bisphenol-A (BPA), and acetaminophen (ACN) measured for ESPA1-2521 elements before and after modification as a function of time. Modification was performed for 0.5 h using 2 mM GMA with the addition of 0.045 mM Triton. The filled and open symbols represent concentrations before and after modification, respectively. CBZ concentration in permeate after modification became undetectably low after one day.

Figure 6 shows the changes in the permeate concentrations of the tested micropollutants over time measured for the GMA-modified and pristine ESPA1-2521 elements. The relatively rapid decrease in the pollutant concentration in the feed over the first six hours of filtration (not shown) could be attributed to sorption, as well as rejection of micropollutants by the membranes. However, after about a day, the permeate concentrations stabilized and steady-state rejection could be measured. It is also clear that, after reaching the steady state, the modified membrane element showed a higher micropollutant rejection than the pristine one (see Figure 6). Table 2 summarizes the solute passages and the hydraulic permeabilities (*L_p_*) measured for the pristine and the modified elements, as the average of the results obtained during the filtration runs. It is seen that the passage of ACN and BPA through the modified ESPA1 element was two and four times lower compared to the pristine one, respectively, and the passage of CBZ dropped to an undetectable level. 

It may be noted that the solute passage, both before and after modification, was highest for ACN, which correlates with its smaller size, compared to the other solutes (Table 1). On the other hand, it does not correlate with the smaller value of logK_ow_, the logarithm of octanol/water partition coefficient (see Table 1). The latter parameter was shown to correlate with the affinity of organic solutes toward the membrane, i.e., the propensity to adsorb or dissolve within the selective layer [5,19,20,21,51]. This suggests that, in the present case, steric effects play a more dominant role than affinity. Previously, we attributed the improved rejection (lower passage) to tightening and better utilization of the polyamide top layer by sealing (“caulking”) less selective areas (“defects”), which should indiscriminately block non-selective passage and improve rejection of all solutes [25,35,38]. It was then anticipated that such sealing will affect larger solutes more strongly, which is indeed observed here. Yet, some effect of affinity is seen as well, while comparing BPA and CBZ. These solutes have a similar size and logK_ow_. However, as phenolic compounds are known to exhibit an exceptionally high affinity and sorption with polyamide layers [51,52], this factor may be responsible for the difference and explain the much lower and more difficult-to-stabilize rejection of BPA compared to CBZ. 

Unfortunately, the improvement in pollutant rejection after modification came at the expense of reduced flux. For the membrane modules modified in this study, the drop in membrane water permeability was significant, when measured with DW (~46%). However for the feed containing micropollutants, the decrease in permeability was more moderate (20–25%, see Table 2), apparently as a result of concentration polarization (see below) and the effect of micropollutants on membrane permeability [51,52,53]. The more significant loss of pure water permeability may then not fully represent the actual drop in performance in realistic operation conditions. Moreover, since the original membrane was a high-flux LPRO membrane, the reduced permeability could still be acceptable and the permeability–selectivity trade-off could compare well with commercial brackish water RO membranes (see below).

Note that the results in Figure 6 could be somewhat biased by differences in concentration polarization, which was supposed to become lower after modification, since, in micropollutant filtration experiments, the flux dropped from about about 36 L/(m^2^ h) for pristine element to 28 L/(m^2^ h) for modified element. However, the mass transfer coefficients evaluated using filtration of NaCl solutions indicated that polarization was, in fact, more significant after modification. The reason is that, after modification, due to lower membrane permeability, a smaller feed flow rate had to be used in order to keep a similar recovery rate for the same feed pressure (20 bar). As a result, concentration polarization factor (CPF) did not exceed 1.4 for pristine elements, but increased to ~3, i.e., about twofold, after modification, despite lower water permeability. This suggests that the drop in pollutant permeability after modification was even more significant than indicated by Figure 6, if the results are corrected for CPF. For instance, the NaCl permeability corrected for CPF dropped by about half after modification, i.e., NaCl rejection increased as well (see below).

**Figure 7:**
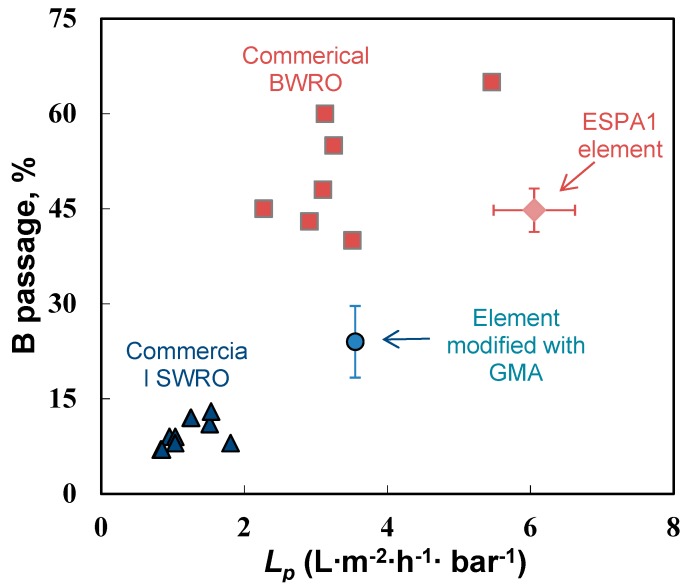
Permeability and boric acid passage of commercial seawater (triangles) and brackish water (squares) reverse osmosis elements, pristine ESPA1 element (diamond), and modified ESPA1 element (circle). Modification conditions: 2 mM GMA with addition of 0.045 mM Triton, modification time 0.5 h.

Membrane elements modified with polyGMA also showed improved boron rejection. Figure 7 shows the boron passage data and the hydraulic permeability obtained for the pristine and the modified ESPA1-2521 modules, along with similar data for some commercial sea water (SW) and brackish water (BW) RO elements compiled from the literature [33,38,54,55,56]. It is seen that the modification of ESPA1 elements resulted in a decrease in boric acid passage, along with a drop in the water permeability. However, compared with the reported performance of commercial BW RO elements, the GMA-modified module still showed a lower passage of boric acid for a similar hydraulic permeability. In addition, the modification produced a significant reduction in the salt passage through the membrane; 0.25–0.5% NaCl passage was measured for the modified module compared to the typical 0.5–1% values of BW RO membranes of similar permeability [39].

## 4. Conclusions

The presented results demonstrate the feasibility of improving rejection of a wide variety of micropollutants in commercial RO membrane elements by in situ modification. While tested with model micropollutant solutions, the modified element showed a moderate (<25%) loss of flux and a significant gain in rejection of all solutes, including organic micropollutants, boric acid, and salt. Following a comparison with reported performance of commercial membranes of similar permeability, the trade-off between the solute passage and water permeability showed that the modified element favorably compared with commercial BW RO elements.

The fact that the improvement was significant for all tested solutes points to the generic nature of the proposed modification. Previously, based on experiments with small coupons, we concluded that the coating layer helps better utilize the high selectivity of the polyamide layer by sealing the tiny defects and indiscriminately blocking non-selective passage of all solutes (“caulking”), rather than by directly rejecting the solutes. Present results demonstrate that this effect may be utilized in the modified elements as well, despite their inherently lower defect rate and better initial performance than small laboratory coupons used in previous studies. The proposed in situ coating procedure based on concentration polarization- and surfactant-enhanced surface polymerization may then be a feasible way to improve rejection of organic and inorganic pollutants and tune membrane performance.

## Figures and Tables

**Table 1:** Model organic micropollutants used and their main characteristics. EDC—endocrine-disrupting compound; PhAC—pharmaceutically active compound.

Characteristics	Bisphenol-A Plastic Additive, EDC	Carbamazepine Drug, PhAC	Acetaminophen Drug, PhAC
Abbreviation	BPA	CBZ	ACN
Molecular weight	228	236.3	151
Solubility in water (mg/L) *	120	18	14,000
Log Kow *	3.32	2.45	0.46
pKa	9.6–10.2 **	13.9 ***	9.4 ***
Chemical structure	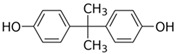	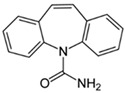	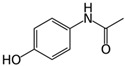

* [20], ** [41], *** [3].

**Table 2:** Passage of carbamazepine (CBZ), bisphenol-A (BPA), and acetaminophen (ACN) and permeability before and after modification of the ESPA1 element for 0.5 h using 2 mM GMA solution with added 0.045 mM Triton; n.d.—not determined; DW—deionized water.

Before/After Modificaton	Solute Passage, %			Hydraulic Permeability *L_p_*, L/m^2^ h bar	
	CBZ	BPA	ACN	DW	CBZ/BPA/ACN Solution
Before modification	0.33 ± 0.06	0.34 ± 0.05	0.7 ± 0.06	6.5 ± 0.05	1.8 ± 0.3
After modification	n.d.	0.09 ± 0.007	0.41 ± 0.03	3.5 ± 0.1	1.4 ± 0.1
